# Correction: Research using population-based administration data integrated with longitudinal data in child protection settings: A systematic review

**DOI:** 10.1371/journal.pone.0303943

**Published:** 2024-05-15

**Authors:** Fadzai Chikwava, Reinie Cordier, Anna Ferrante, Melissa O’Donnell, Renée Speyer, Lauren Parsons

The ORCID iDs are missing for the second, third, and fourth author. Please see the authors’ respective ORCID iDs here:

- Author Reinie Cordier’s ORCID iD is: 0000-0002-9906-5300 (https://orcid.org/0000-0002-9906-5300).
- Author Anna Ferrante’s ORCID iD is: 0000-0002-9479-7908 (https://orcid.org/0000-0002-9479-7908).
- Author Melissa O’Donnell’s ORCID iD is: 0000-0003-2440-3667 (https://orcid.org/0000-0003-2440-3667).

In the PDF version, there are a number of errors in the headings for Table 2. Please see the complete, correct Table 2 here.

**Table 2:** Characteristics of included studies.

**Author (Year)**	**Country**	**Aims/ Objectives**	**Research Area**	**Child Protection Contact (CPC) vs. OHC**	**Administrative Data Source**	**Number of administrative datasets (Deterministic/ Probabilistic Linkage)**	**Linkage Quality (Yes/ No)**
**Egulend et al. (2009)**	Denmark	To identify problems among children in foster and residential care compared to in home care children, and to all non-welfare children of the same age, and to analyse factors associated with mental health problems in children in out-of-home care	Mental Health	OHC	1.National Health Register; 2.Psychiatric Research Register 3.Child Protection Register	2 (Deterministic)	No
**Hansson et al. (2018)**	Sweden	To describe and discuss differences between children placed in OHC and non-OHC children in the Swedish compulsory school, with respect to special needs education, school mobility and academic achievement.	Education	OHC	Statistics Sweden	1 (NR)	No
**Kisely et al. (2019)**	Australia	To examine whether notified and/or substantiated child maltreatment is associated with the prevalence and persistence of smoking in early adulthood	Drugs & Alcohol	CPC	Queensland Department of Families, Youth and Community Care (DFYCC)	1 (Deterministic)	No
**Kisely et al. (2018)**	Australia	To examine, using a prospective record-linkage analysis, whether substantiated child maltreatment is associated with adverse psychological outcomes in early adulthood.	Mental Health	CPC	Queensland Department of Families, Youth and Community Care (DFYCC)	1 (Deterministic)	No
**Kisely et al. (2019)**	Australia	To study the association of different types of child maltreatment with alcohol use disorders at 21 years of age	Drugs & Alcohol	CPC	Queensland Department of Families, Youth and Community Care (DFYCC)	1 (Deterministic)	No
**Olsen et al. (2018)**	Denmark	To investigate the association for children in OHC and non-OHC peers between school change in lower secondary school and two educational outcomes: (1) self-perceived academic abilities at age 15 and (2) staying-on rates in upper secondary school at age 18	Education	OHC	Danish Register Data	1 (Deterministic)	No
**Author (Year)**	**Country**	**Aims/ Objectives**	**Research Area**	**Child Protection Contact (CPC) vs. OHC**	**Administrative Data Source**	**Number of administrative datasets (Deterministic/ Probabilistic Linkage)**	**Linkage Quality (Yes/ No)**
**Parrish et al. (2016)**	USA	To determine the predictive relationship between a maternal pre-birth self-reported history of intimate partner violence (IPV) and any post-birth reported allegation to Child Protective Services (CPS) by age 2	Domestic violence	CPC	Alaska’s Child Protective Services Agency Register	1 (Probabilistic)	No
**Parrish et al. (2017)**	USA	A description of the creation of the (ALCANLink) project and the benefit of the ALCANLink methodology by documenting the bias in incidence and hazard ratios that can arise in birth cohort linkage studies due to incomplete data linkages, non-linkage assumptions, and single source outcome ascertainment	Child protection	CPC	1. Vital records; 2. Child death review; 3. Alaska Permanent Fund Dividend (PFD) records	3 (Deterministic & Probabilistic)	Yes
**Raghavan et al. (2017)**	USA	To quantify the magnitude of non-ascertainment bias, develop a profile of children who are at greatest risk for non-ascertainment,	Health insurance	OHC	1.Medicaid Analytic eXtract (MAX) Research Data Assistance Centre; 2.Child Welfare Agency	1 (Deterministic)	Yes
**Sidebotham et al. (2000)**	UK	A study of patterns of child abuse and factors that may affect risk in a pre-school population	Child protection	CPC	Avon Social Services Child Protection Register	1 (NR)	No
**Sidebotham et al. (2003)**	UK	To determine characteristics of children that may predispose to maltreatment.	Child protection	CPC	Avon Social Services Child Protection Register	1 (NR)	No
**Sidebotham et al. (2006)**	UK	to analyse the multiple factors affecting risk of abuse in young children within a comprehensive theoretical framework	Child protection	CPC	Avon Social Services Child Protection Register	1 (NR)	No
							
**Author (Year)**	**Country**	**Aims/ Objectives**	**Research Area**	**Child Protection Contact (CPC) vs. OHC**	**Administrative Data Source**	**Number of administrative datasets (Deterministic/ Probabilistic Linkage)**	**Linkage Quality (Yes/ No)**
**Sidebotham et al. (2002)**	UK	To determine risk factors for child maltreatment within the socio-economic environment of a contemporary UK child population	Child protection	CPC	Avon Social Services Child Protection Register	1 (NR)	No
**Teyhan et al. (2019)**	UK	To use record linkage of birth cohort and administrative data to study educational outcomes of children who are looked-after (in public care) and in need (social services involvement), and examine the role of early life factors.	Education	OHC	1. Children Looked-After (CLA) Data Return; 2. Children in Need (CIN) Census; 3. National Pupil Database	3 (NR)	No
**Austin et al. (2019)**	USA	Identify longitudinal trajectory classes of CPS contact among Alaska Native (AN/AI) and non-Native (NN) children and examine preconception and prenatal risk factors associated with identified classes	Child protection	CPC	1. Alaska Office of Children’s Services (OCS); 2. Alaska Child Death Review; 3. Death certificate files; 4. Alaska Dept. of Revenue	4 (NR)	No
**Austin et al. (2018)**	USA	To use multiple novel data sources and time-to event analysis to examine preconception and prenatal predictors of time to first contact with CPS among a representative sample of Alaska children.	Child protection	CPC	1. Alaska Office of Children’s Services (OCS); 2. Alaska Child Death Review; 3. Death certificate files; 4. Alaska Dept. of Revenue 4. Geographic census classification data 6. Alaska Birth Defects Registry	6 (NR)	No
**Hansson et al. (2020)**	Sweden	To investigate the effects of school mobility on academic achievements for OHC-children as well as for NOHC-children.	Education	OHC	Statistics Sweden: Child Welfare Register	1 (NR)	No
**Abajobir et al. (2017)**	Australia	Examine the association between different types of substantiated child maltreatment and self-reported psychotic experiences as measured by the Young Adult Self-Report (YASR) items and the Peter’s Delusions Inventory (PDI) using data from a large population-based birth cohort study.	Mental Health	CPC	Queensland Department of Families, Youth and Community Care (DFYCC)	1 (Deterministic)	No
**Author (Year)**	**Country**	**Aims/ Objectives**	**Research Area**	**Child Protection Contact (CPC) vs. OHC**	**Administrative Data Source**	**Number of administrative datasets (Deterministic/ Probabilistic Linkage)**	**Linkage Quality (Yes/ No)**
**Abajobir et al. (2017)**	Australia	Examine the effect on QoL of multiple forms of substantiated child maltreatment controlling for selected potential confounders and/covariates, and concurrent depressive symptoms.	Mental Health	CPC	Queensland Department of Families, Youth and Community Care (DFYCC)	1 (Deterministic)	No
**Abajobir et al. (2016)**	Australia	This study examines whether distinct types of childhood maltreatment differentially predict different forms of intimate partner violence	Domestic violence	CPC	Queensland Department of Families, Youth and Community Care (DFYCC)	1 (Deterministic)	No
**Abajobir et al. (2016)**	Australia	This study investigates the association between exposure to prospectively-substantiated childhood maltreatment between 0 to 14 years of age and lifetime cannabis use, abuse and dependence reported at 21 years	Drugs & alcohol	CPC	Queensland Department of Families, Youth and Community Care (DFYCC)	1 (Deterministic)	No
**Abajobir et al. (2017)**	Australia	Determine the association between substantiated childhood maltreatment and injecting drug use	Drugs & Alcohol	CPC	Queensland Department of Families, Youth and Community Care (DFYCC)	1 (Deterministic)	No
**Strathean et al. (2009)**	Australia	Explored whether breastfeeding may protect against maternally-perpetrated child maltreatment.	Child protection	CPC	Queensland Department of Families, Youth and Community Care (DFYCC)	1 (Deterministic)	No
**Mills et al. (2013)**	Australia	To examine whether notified child maltreatment is associated with adverse psychological outcomes in adolescence, and whether differing patterns of psychological outcome are seen depending on the type of maltreatment.	Mental Health	CPC	Queensland Department of Families, Youth and Community Care (DFYCC)	1 (Deterministic)	No
**Author (Year)**	**Country**	**Aims/ Objectives**	**Research Area**	**Child Protection Contact (CPC) vs. OHC**	**Administrative Data Source**	**Number of administrative datasets (Deterministic/ Probabilistic Linkage)**	**Linkage Quality (Yes/ No)**
**Mills et al. (2016)**	Australia	Investigate the incidence of CSA in the same birth cohort using both retrospective self-report and prospective government agency notification, and examine the psychological outcomes in young adulthood.	Mental Health	CPC	Queensland Department of Families, Youth and Community Care (DFYCC)	1 (Deterministic)	No
**Mills et al. (2014)**	Australia	This study examines whether child maltreatment experience predicts adolescent tobacco and alcohol use. The secondary question was whether specific patterns of types of maltreatment were associated with alcohol and/or tobacco use.	Drugs & alcohol	CPC	Queensland Department of Families, Youth and Community Care (DFYCC)	1 (Deterministic)	No
**Mills et al. (2019)**	Australia	to investigate whether child maltreatment is associated with adverse outcomes in cognitive function, high school completion and employment by the age of 21	Education	CPC	Queensland Department of Families, Youth and Community Care (DFYCC)	1 (Deterministic)	No
**Mills et al. (2017)**	Australia	To investigate whether: (1) child maltreatment is associated with life-time cannabis use, early-onset cannabis use, daily cannabis use and DSM-IV cannabis abuse in young adulthood; and (2) behaviour problems, tobacco use and alcohol use at age 14 are associated with cannabis use.	Drugs & Alcohol	CPC	Queensland Department of Families, Youth and Community Care (DFYCC)	1 (Deterministic)	No
**Parrish et al. (2011)**	Australia	To assess the utility of combining PRAMS data with child protective services (CPS) records to identify risk factors associated with Protective Services Reports (PSR) suggestive of child maltreatment	Child protection	CPC	Alaska’s Child Protective Services Agency Register	1 (Probabilistic)	Yes
**Raghavan et al. (2012)**	USA	To estimate the amount of Medicaid expenditures incurred from the purchase of psychotropic drugs–the primary drivers of mental health expenditures among children in the child welfare system	Health insurance	CPC	1.Medicaid Analytic eXtract (MAX) Research Data Assistance Centre; 2.Child Welfare Agency	1 (Deterministic & Probabilistic)	Yes

**Author (Year)**	**Name of Longitudinal Study**	**Study Period**	**Sampling Method**	**Study Population**				**Waves in the study: (Age: sample size)**	**Wave reported: (Age: Sample Size)**
				**Age at Baseline**	**Year of birth**	**Gender-Males (%)**	**Cohort size at Baseline**		
**Egulend et al. (2009)**	Danish longitudinal survey of children (DALSC)	1995–2007	NR	Birth	1995	NR	1. Non-CPC (6,000); 2. OHC (1,072); 3. In-home care (1,457)	Wave 1, Baseline: (4 months, n = 6,622); Wave 2: (3.5 years, n = 6,622); Wave 3: (7 years, n = 7,198); Wave 4: (11 years, n = 8,225); Wave 5: (15 years, n = 7,132)	Wave 4: (11 years, Non-welfare children n = 5,242; OHC: n = 433; In-home care: n = 95)
**Hansson et al. (2018)**	Swedish longitudinal Evaluation Through Follow-up (ETF) project	1971–2001	Stratified systematic sampling	9 years	1972; 1977; 1982; 1987; 1992	NR	(4,500–12,000)* 5 Cohorts	1948 Cohort: (12 years, n = 12,000); 1953 Cohort: (12 years, n = 9,000); 1967 Cohort: (12 years, n = 9,000); 1972 Cohort: (9 & 12 years, n = 9,000); 1977 Cohort: (9 & 12 years, n = 4,500); 1982 Cohort: (12 years, n = 9,000); 1987 Cohort: (15 years, n = 9,000); 1992 Cohort: (9 years, n = 9,000)	Wave 1, Baseline (7 years; n = N/A); Wave 2: (9 years; Pooled Data from 5 Cohorts (non-OHC: n = 40,107; OHC: n = 1,482)
**Kisely et al. (2019)**	The Mater-University Study of Pregnancy (MUSP)	1981–2004	NR	Birth	1981–1983	47%	7,223 Mother & Child pairs	Wave 1, Baseline: (Mother and child dyads at birth, n = 7,223); Wave 2: (6 months: n = 6,720); Wave 3: (5 years: n = 5,308); Wave 4: (14 years: n = 5,216); Wave 5: (21 years: n = 3,805); Wave 6: (30 years: n = 2,904)	Wave 1, Baseline: (Mother and child dyads at birth, n = 7,223); Wave 4 (14 years: n = NR); Wave 5 (21 years: n = 3,758 & subset n = 2,548)
**Author (Year)**	**Name of Longitudinal Study**	**Study Period**	**Sampling Method**	**Study Population**				**Waves in the study: (Age: sample size)**	**Wave reported: (Age: Sample Size)**
				**Age at Baseline**	**Year of birth**	**Gender-Males (%)**	**Cohort size at Baseline**		
**Kisely et al. (2018)**	The Mater-University Study of Pregnancy (MUSP)	1981–2004	NR	Birth	1981–1983	53%	7,223 Mother & Child pairs	Wave 1, Baseline: (Mother and child dyads at birth, n = 7,223); Wave 2: (6 months: n = 6,720); Wave 3: (5 years: n = 5,308); Wave 4: (14 years: n = 5,216); Wave 5: (21 years: n = 3,805); Wave 6: (30 years: n = 2,904)	Wave 1, Baseline: (Mother and child dyads at birth: n = 7,223); Wave 5 (21 years: n = 3,778)
**Kisely et al. (2019)**	The Mater-University Study of Pregnancy (MUSP)	1981–2004	NR	Birth	1981–1983	47%	7,223 Mother & Child pairs	Wave 1, Baseline: (Mother and child dyads at birth: n = 7,223); Wave 2: (6 months: n = 6,720); Wave 3: (5 years: n = 5,308); Wave 4: (14 years: n = 5,216); Wave 5: (21 years: n = 3,805); Wave 6: (30 years: n = 2,904)	Wave 1, Baseline: (Mother and child dyads at birth: n = 7,223); Wave 5 (21 years: n = 3,762)
**Olsen et al. (2018)**	Danish longitudinal survey of children (DALSC)	1995–2011	NR	Birth	1995	53%	907 OHC; 5,900 non-OHC	Wave 1, Baseline: (4 months, n = 6,622); Wave 2: (3.5 years: n = 6,622); Wave 3: (7 years: n = 7,198); Wave 4: (11 years: n = 8,225); Wave 5: (15 years: n = 7,132); Wave 6: (18 years: n = 5,139)	Wave 1, Baseline: (Birth, OHC: n = 907, non-OHC: n = 5,900); Wave 5: (15 years: OHC: n = 169, non-OHC: n = 4,568); Wave 6: (18 years: OHC: n = 817, non-OHC: n = 4,322)
**Parrish et al. (2016)**	Alaska Pregnancy Risk Assessment Monitoring System (PRAMS)	2009–2014	Stratified systematic sampling	Birth	2009–2010	NR	2,389	1990–2016 Cohorts: (Annual sample sizes per state range from about 1000 to 3000 women)	Wave 1: (Birth-2 years: n = 2,389)
**Parrish et al. (2017)**	Alaska Pregnancy Risk Assessment Monitoring System (PRAMS)	2009–2014	Stratified systematic sampling	Birth	2009–2011	NR	1,235	1990–2016 Cohorts: (Annual sample sizes per state range from about 1000 to 3000 women)	Wave 1: (Birth: n = 1,235)
**Raghavan et al. (2017)**	National Survey of Child and Adolescent Well-Being (NSCAW)	1999–2003	NR	NR	NR	NR	Child Protection Contact (CPC) (5,501); Long term foster care placement (LTFC) (727)	Wave 1: (Birth: n = 6,228); Wave 2: (9 years: n = 5,873); Wave 3: (14 years: n = NR)	Pooled (Wave 1-wave 3) sample: (CPS: n = 2,309, LTFC: n = 423)
**Author (Year)**	**Name of Longitudinal Study**	**Study Period**	**Sampling Method**	**Study Population**				**Waves in the study: (Age: sample size)**	**Wave reported: (Age: Sample Size)**
				**Age at Baseline**	**Year of birth**	**Gender-Males (%)**	**Cohort size at Baseline**		
**Sidebotham et al. (2000)**	The Avon Longitudinal Study of Parents and Children (ALSPAC)	1991–1998	NR	Pre-birth	1991–1992	NR	14,451	Wave 1: (Pre-birth: n = 14,893); Wave 2: (1 month: n = 14,256); Wave 3: (6–8 months: n = 11,194, Partner = 6,861); Wave 4: (18 months: n = 10,750); Wave 5: (21 months: n = 10,323); Wave 6: (30 months: n = 10,289); Wave 7: (33 months: n = 9,635)	Wave 3: (8 months, n = 11,194, Partner: n = 6,861); Wave 4: (18 months, n = 10,750); Wave 5: (21 months, n = 10,323); Wave 6: (30 months, n = 10,289); Wave 7: (33 months, n = 9,635)
**Sidebotham et al. (2003)**	The Avon Longitudinal Study of Parents and Children (ALSPAC)	1991–1998	NR	1 month	1991–1992	(56% registered & 52% non-registered)	14,256	Wave 1: (Pre-birth: n = 14,893); Wave 2: (1 month: n = 14,256); Wave 3: (6–8 months: n = 11,194, Partner = 6,861); Wave 4: (18 months: n = 10,750); Wave 5: (21 months: n = 10,323); Wave 6: (30 months: n = 10,289); Wave 7: (33 months: n = 9,635)	Wave 2: (1 month, n = 14,256); Wave 6: (30 months, n = 115 registered vs n = 14,105 non-registered children)
**Sidebotham et al. (2006)**	The Avon Longitudinal Study of Parents and Children (ALSPAC)	1991–1998	NR	Pre-birth	1991–1992	NR	14,256	Wave 1: (Pre-birth: n = 14,893); Wave 2: (1 month: n = 14,256); Wave 3: (6–8 months: n = 11,194, Partner = 6,861); Wave 4: (18 months: n = 10,750); Wave 5: (21 months: n = 10,323); Wave 6: (30 months: n = 10,289); Wave 7: (33 months: n = 9,635)	Wave 2: (One month: n = 14,256); Wave 7: (36 months: n = NR)
**Author (Year)**	**Name of Longitudinal Study**	**Study Period**	**Sampling Method**	**Study Population**				**Waves in the study: (Age: sample size)**	**Wave reported: (Age: Sample Size)**
				**Age at Baseline**	**Year of birth**	**Gender-Males (%)**	**Cohort size at Baseline**		
**Sidebotham et al. (2002)**	The Avon Longitudinal Study of Parents and Children (ALSPAC)	1991–1998	NR	Pre-birth	1991–1992	52%	14,256	Wave 1: (Pre-birth: n = 14,893); Wave 2: (1 month: n = 14,256); Wave 3: (6–8 months: n = 11,194, Partner = 6,861); Wave 4: (18 months: n = 10,750); Wave 5: (21 months: n = 10,323); Wave 6: (30 months: n = 10,289); Wave 7: (33 months: n = 9,635)	Wave 2: (One month: n = 14,256); Wave 3: (8 months: n = 11,194); Wave 5: (21 months: n = 10,323); Wave 7: (33 months: n = 9,635)
**Teyhan et al. (2019)**	The Avon Longitudinal Study of Parents and Children (ALSPAC)	1991–2009	NR	Pre-birth	1991–1992	(50% (No CLA/CIN); 48% CIN; 51% CLA)	14,868	Wave 1: (Pre-birth: n = 14,893); Wave 2: (1 month: n = 14,256); Wave 3: (6–8 months: n = 11,194, Partner = 6,861); Wave 4: (18 months: n = 10,750); Wave 5: (21 months: n = 10,323); Wave 6: (30 months: n = 10,289); Wave 7: (33 months: n = 9,635)	Wave 3: (1 year: n = 13,988); Wave 8: (7–18 years, Booster: n = 718); Wave 9: (>18 years, Booster: n = 183)
**Austin et al. (2019)**	Alaska Longitudinal Child Abuse and Neglect Linkage (ALCANLink) project & PRAMS	2009–2014	Stratified systematic sampling	Birth	2009–2011	(53% AN & 49% NN)	AN (1,257); NN (2,102)	1990–2016 Cohorts: (Birth, n = 1,000–3,000)	Wave 1: (Birth -5/6 years)
**Austin et al. (2018)**	Alaska Longitudinal Child Abuse and Neglect Linkage (ALCANLink) project & PRAMS	2009–2015	Stratified systematic sampling	Birth	2009–2011	51%	3,549	1990–2016 Cohorts: (Birth, n = 1,000–3,000)	Wave 1 (Birth -5/6 years)
**Author (Year)**	**Name of Longitudinal Study**	**Study Period**	**Sampling Method**	**Study Population**				**Waves in the study: (Age: sample size)**	**Wave reported: (Age: Sample Size)**
				**Age at Baseline**	**Year of birth**	**Gender-Males (%)**	**Cohort size at Baseline**		
**Hansson et al. (2020)**	Swedish longitudinal Evaluation Through Follow-up (ETF) project	NR	Stratified systematic sampling	9 years	1972; 1977; 1982; 1987; 1992	NR	(4,500–12,000)* 5 Cohorts	1948 Cohort: (12 years, n = 12,000); 1953 Cohort: (12 years, n = 9,000); 1967 Cohort: (12 years, n = 9,000); 1972 Cohort: (9 & 12 years, n = 9,000); 1977 Cohort: (9 & 12 years, n = 4,500); 1982 Cohort: (12 years, n = 9,000); 1987 Cohort: (15 years, n = 9,000); 1992 Cohort: (9 years, n = 9,000)	Wave 2: (9 years, n = NR); Wave 3: (12 years, n = NR)
**Abajobir et al. (2017)**	The Mater-University Study of Pregnancy (MUSP)	1981–2004	NR	Birth	1981–1983	47%	7,223 Mother & Child pairs	Wave 1, Baseline: (Mother and child dyads at birth: n = 7,223); Wave 2: (6 months: n = 6,720); Wave 3: (5 years: n = 5,308); Wave 4: (14 years: n = 5,216); Wave 5: (21 years: n = 3,805); Wave 6: (30 years: n = 2,904)	Wave 1, Baseline: (Mother and child dyads at birth, n = 7,223); Wave 3: 5 years; Wave 4 (14 years: n = NR); Wave 5 (21 years: n = 3,752)
**Abajobir et al. (2017)**	The Mater-University Study of Pregnancy (MUSP)	1981–2004	NR	Birth	1981–1983	50%	7,223 Mother & Child pairs	Wave 1, Baseline: (Mother and child dyads at birth: n = 7,223); Wave 2: (6 months: n = 6,720); Wave 3: (5 years: n = 5,308); Wave 4: (14 years: n = 5,216); Wave 5: (21 years: n = 3,805); Wave 6: (30 years: n = 2,904)	Wave 1, Baseline: (Mother and child dyads at birth, n = 7,223); Wave 3: (5 years: n = NR); Wave 4 (14 years: n = NR); Wave 5 (21 years: n = 3,730)
**Author (Year)**	**Name of Longitudinal Study**	**Study Period**	**Sampling Method**	**Study Population**				**Waves in the study: (Age: sample size)**	**Wave reported: (Age: Sample Size)**
				**Age at Baseline**	**Year of birth**	**Gender-Males (%)**	**Cohort size at Baseline**		
**Abajobir et al. (2016)**	The Mater-University Study of Pregnancy (MUSP)	1981–2004	NR	Birth	1981–1983	45%	7,223 Mother & Child pairs	Wave 1, Baseline: (Mother and child dyads at birth: n = 7,223); Wave 2: (6 months: n = 6,720); Wave 3: (5 years: n = 5,308); Wave 4: (14 years: n = 5,216); Wave 5: (21 years: n = 3,805); Wave 6: (30 years: n = 2,904)	Wave 1, Baseline: (Mother and child dyads at birth: n = 7,223); Wave 4 (14 years: n = NR); Wave 5 (21 years: n = 3,322)
**Abajobir et al. (2016)**	The Mater-University Study of Pregnancy (MUSP)	1981–2004	NR	Birth	1981–1983	48%	7,223 Mother & Child pairs	Wave 1, Baseline: (Mother and child dyads at birth: n = 7,223); Wave 2: (6 months: n = 6,720); Wave 3: (5 years: n = 5,308); Wave 4: (14 years: n = 5,216); Wave 5: (21 years: n = 3,805); Wave 6: (30 years: n = 2,904)	Wave 1, Baseline: (Mother and child dyads at birth: n = 7,223); Wave 4 (14 years: n = NR); Wave 5 (21 years: n = 2,526)
**Abajobir et al. (2017)**	The Mater-University Study of Pregnancy (MUSP)	1981–2004	NR	Birth	1981–1983	47%	7,223 Mother & Child pairs	Wave 1, Baseline: (Mother and child dyads at birth: n = 7,223); Wave 2: (6 months: n = 6,720); Wave 3: (5 years: n = 5,308); Wave 4: (14 years: n = 5,216); Wave 5: (21 years: n = 3,805); Wave 6: (30 years: n = 2,904)	Wave 1, Baseline: (Mother and child dyads at birth, n = 7,223); Wave 5: (21 years: n = 3,750)
**Strathean et al. (2009)**	The Mater-University Study of Pregnancy (MUSP)	1981–2000	NR	Birth	1981–1983	52%	7,223 Mother & Child pairs	Wave 1, Baseline: (Mother and child dyads at birth: n = 7,223); Wave 2: (6 months: n = 6,720); Wave 3: (5 years: n = 5,308); Wave 4: (14 years: n = 5,216); Wave 5: (21 years: n = 3,805); Wave 6: (30 years: n = 2,904)	Wave 1, Baseline: (Mother and child dyads at birth, n = 7,223); Wave 2: (6 months: n = 6,621); Wave 4: (15 years: n = 5,890)
**Author (Year)**	**Name of Longitudinal Study**	**Study Period**	**Sampling Method**	**Study Population**				**Waves in the study: (Age: sample size)**	**Wave reported: (Age: Sample Size)**
				**Age at Baseline**	**Year of birth**	**Gender-Males (%)**	**Cohort size at Baseline**		
**Mills et al. (2013)**	The Mater-University Study of Pregnancy (MUSP)	1981–2000	NR	Birth	1981–1983	52%	7,223 Mother & Child pairs	Wave 1, Baseline: (Mother and child dyads at birth: n = 7,223); Wave 2: (6 months: n = 6,720); Wave 3: (5 years: n = 5,308); Wave 4: (14 years: n = 5,216); Wave 5: (21 years: n = 3,805); Wave 6: (30 years: n = 2,904)	Wave 1, Baseline: (Mother and child dyads at birth, n = 7,223); Wave 4: (14 years: n = 5,172)
**Mills et al. (2016)**	The Mater-University Study of Pregnancy (MUSP)	1981–2004	NR	Birth	1981–1983	52%	7,223 Mother & Child pairs	Wave 1, Baseline: (Mother and child dyads at birth: n = 7,223); Wave 2: (6 months: n = 6,720); Wave 3: (5 years: n = 5,308); Wave 4: (14 years: n = 5,216); Wave 5: (21 years: n = 3,805); Wave 6: (30 years: n = 2,904)	Wave 1, Baseline: (Mother and child dyads at birth, n = 7,223); Wave 5: (21 years: n = 3,739)
**Mills et al. (2014)**	The Mater-University Study of Pregnancy (MUSP)	1981–2000	NR	Birth	1981–1983	52%	7,223 Mother & Child pairs	Wave 1, Baseline: (Mother and child dyads at birth: n = 7,223); Wave 2: (6 months: n = 6,720); Wave 3: (5 years: n = 5,308); Wave 4: (14 years: n = 5,216); Wave 5: (21 years: n = 3,805); Wave 6: (30 years: n = 2,904)	Wave 1, Baseline: (Mother and child dyads at birth: n = 7,223); Wave 4: (14 years: n = 5,200)
**Mills et al. (2019)**	The Mater-University Study of Pregnancy (MUSP)	1981–2004	NR	Birth	1981–1983	NR	7,223 Mother & Child pairs	Wave 1, Baseline: (Mother and child dyads at birth: n = 7,223); Wave 2: (6 months: n = 6,720); Wave 3: (5 years: n = 5,308); Wave 4: (14 years: n = 5,216); Wave 5: (21 years: n = 3,805); Wave 6: (30 years: n = 2,904)	Wave 1, Baseline: (Mother and child dyads at birth: n = 7,223); Wave 5: (21 years: n = 3,778)
**Author (Year)**	**Name of Longitudinal Study**	**Study Period**	**Sampling Method**	**Study Population**				**Waves in the study: (Age: sample size)**	**Wave reported: (Age: Sample Size)**
				**Age at Baseline**	**Year of birth**	**Gender-Males (%)**	**Cohort size at Baseline**		
**Mills et al. (2017)**	The Mater-University Study of Pregnancy (MUSP)	1981–2004	NR	Birth	1981–1983	47%	7,223 Mother & Child pairs	Wave 1, Baseline: (Mother and child dyads at birth: n = 7,223); Wave 2: (6 months: n = 6,720); Wave 3: (5 years: n = 5,308); Wave 4: (14 years: n = 5,216); Wave 5: (21 years: n = 3,805); Wave 6: (30 years: n = 2,904)	Wave 1, Baseline (Mother and child dyads at birth: n = 7,223); Wave 4: (14 years: n = NR); Wave 5: (21 years: n = 3,778)
**Parrish et al. (2011)**	Alaska Pregnancy Risk Assessment Monitoring System (PRAMS)	1997–2004	Stratified systematic sampling	Birth	1997–1999	48%	5, 421	1990–2016 Cohorts: (Annual sample sizes per state range from about 1000 to 3000 women)	Wave 1, Baseline (Birth: n = 5,421); Wave 2: (48 months: n = 4,217)
**Raghavan et al. (2012)**	National Survey of Child and Adolescent Well-Being (NSCAW)	1999–2003	NR	2 years	NR	48%	NSCAW (2,831); Matched child observations (2,821)	Wave 1: (Birth: n = 6,228); Wave 2: (9 years: n = 5,873); Wave 3: (14 years: n = NR)	Pooled (Wave 1-wave 4): n = 5,652

**Author (Year)**	**Timeframe between reported waves (months)**	**Outcome Measures**		**Missing data (Yes/ No)**	**Attrition rate**	**Described attrition (Yes/No)**	**Corrected attrition (Yes/No)**	**Attrition analysis (Yes/No)**	**Selection bias (Yes/ No)**	**Sensitivity analysis Yes/No)**
		**Standardized**	**Non-standardized**							
**Egulend et al. (2009)**	36 months	1. Strengths and Difficulties screening (SDQ) for mental health 2. ICD-10 Psychiatric diagnosis	1. School performance and satisfaction; 2. Leisure activities	Yes	NR	Yes	Yes	No	No	No
**Hansson et al. (2018)**	Waves 1–2 = 24 months	Cognitive Test Scores	Academic achievement	Yes	NR	No	Yes	No	Yes	No
**Kisely et al. (2019)**	(Waves 1–4 = 168 months); Waves 4–5 = 84 months)	1. WHO (CIDI-DSM-IV) scale) for Nicotine use, dependence & withdrawal; 2. Depression (CES-D) scale	1. Prevalence of smoking; 2. Persistent smoking	Yes	48%	Yes	Yes	Yes	No	Yes
**Kisely et al. (2018)**	(Waves 1–5 = 252 months)	1. Centre for Epidemiological Studies-Depression scales (CES-D) 2. Achenbach Youth Self-Report (YASR) scale; 3. WHO (CIDI-DSM-IV) scale	None	Yes	48%	Yes	Yes	Yes	No	Yes
**Kisely et al. (2019)**	(Waves 1–5 = 252 months)	WHO (CIDI-DSM-IV) scale for alcohol use and dependence	Alcohol use in the last month	Yes	48%	Yes	Yes	Yes	No	No
**Olsen et al. (2018)**	(Waves 1–2 = 180 months); Waves 2–3 = 36 months)	None	1. Self-perceived academic ability (SAA) 2. Staying-on rates	Yes	NR	Yes	No	Yes	No	No
**Parrish et al. (2016)**	N/A	None	Maltreatment report to Child Protective Services	Yes	N/A	No	No	No	No	Yes
**Parrish et al. (2017)**	N/A	None	Child maltreatment	Yes	NR	Yes	Yes	Yes	Yes	No
**Raghavan et al. (2017)**	Wave 1- Wave 3 = 36 months	None	Ascertainment of foster care status	Yes	NR	No	No	No	Yes	No
**Sidebotham et al. (2000)**	(Waves 3–4 = 10 months); (Waves 4–5 = 3 months); (Waves 5–6 = 9 months); (Waves 6–7 = 3 months)	None	Child abuse investigations and registrations	No	NR	No	No	No	No	No
**Sidebotham et al. (2003)**	(Waves 2–6 = 29 months)	None	Child protection registration	Yes	NR	Yes	No	No	Yes	No
**Author (Year)**	**Timeframe between reported waves (months)**	**Outcome Measures**		**Missing data (Yes/ No)**	**Attrition rate**	**Described attrition (Yes/No)**	**Corrected attrition (Yes/No)**	**Attrition analysis (Yes/No)**	**Selection bias (Yes/ No)**	**Sensitivity analysis Yes/No)**
		**Standardized**	**Non-standardized**							
**Sidebotham et al. (2006)**	Wave 2–7: 35 months	None	1. Investigation for suspected maltreatment; 2. Registration on the child protection register	Yes	NR	Yes	No	No	Yes	No
**Sidebotham et al. (2002)**	(Waves 2–3 = 7 months); (Waves 3–5 = 13 months); (Waves 5–7 = 12 months)	None	Child abuse registration	Yes	NR	No	No	No	Yes	No
**Teyhan et al. (2019)**	(Waves 3–8 = 84 months); (Waves 8–9 = 132 months)	None	1. Educational attainment; 2. Persistent absence from school; 3. Special educational needs (SEN) status; 4. School Mobility	Yes	NR	No	No	No	No	Yes
**Austin et al. (2019)**	Wave 1 (5/6 years)	None	Child Protective Service Contact	Yes	NR	No	No	No	No	No
**Austin et al. (2018)**	Wave 1 (5/6 years)	None	Age at first CP contact	Yes	NR	No	No	No	No	Yes
**Hansson et al. (2020)**	Waves 2–3 = 36 months	None	Cognitive ability	Yes	NR	No	No	No	No	No
**Abajobir et al. (2017)**	(Waves 1–2 = 6 months); (Waves 2–3 = 54 months); (Waves 3–4 = 108 months); (Waves 4–5 = 84 months)	1. Achenbach’s YASR Behaviour Checklist (Auditory & Visual Hallucinations); 2. Peter’s Delusional Inventory (PDI); 3. WHO (CIDI-DSM-IV) scale for diagnoses of psychosis	None	Yes	48%	Yes	Yes	Yes	Yes	Yes
**Abajobir et al. (2017)**	(Waves 1–2 = 6 months); (Waves 2–3 = 54 months); (Waves 3–4 = 108 months); (Waves 4–5 = 84 months)	1. Achenbach’s Young Adult Self-Report (YASR) Behaviour Checklist (4 items); 2. Centre for Epidemiological Studies Depression Scale (CES-D)	QoL Self Report (Happy/ Satisfaction scales)	Yes	48%	Yes	Yes	Yes	No	No
**Author (Year)**	**Timeframe between reported waves (months)**	**Outcome Measures**		**Missing data (Yes/ No)**	**Attrition rate**	**Described attrition (Yes/No)**	**Corrected attrition (Yes/No)**	**Attrition analysis (Yes/No)**	**Selection bias (Yes/ No)**	**Sensitivity analysis Yes/No)**
		**Standardized**	**Non-standardized**							
**Abajobir et al. (2016)**	(Waves 1–2 = 6 months); (Waves 2–3 = 54 months); (Waves 3–4 = 108 months); (Waves 4–5 = 84 months)	1. Composed abuse scale (CAS) 2. Child Behaviour Checklist (CBCL) 3. Life events scale; 4. Conflict tactics scale	None	Yes	54%	Yes	Yes	Yes	No	Yes
**Abajobir et al. (2016)**	(Waves 1–2 = 6 months); (Waves 2–3 = 54 months); (Waves 3–4 = 108 months); (Waves 4–5 = 84 months)	WHO (CIDI-DSM-IV) scale for Lifetime cannabis abuse and dependence	Early age of onset of cannabis abuse	Yes	65%	Yes	Yes	Yes	Yes	No
**Abajobir et al. (2017)**	(Waves 1–5 = 252 months)	Depression: Delusions-Symptoms-States Inventory scale (DSSI)	Ever injected illicit drugs	Yes	48%	Yes	Yes	Yes	Yes	Yes
**Strathean et al. (2009)**	(Waves 1–3 = 6 months); Waves 3–4 = 174 months)	Depression: Delusions-Symptoms-States Inventory scale (DSSI)	Child maltreatment	Yes	18%	Yes	Yes	Yes	Yes	Yes
**Mills et al. (2013)**	(Waves 1–4 = 168 months)	Achenbach Youth Self-Report (YSR) questionnaires	None	Yes	28%	Yes	No	Yes	No	Yes
**Mills et al. (2016)**	(Waves 1–5 = 252 months)	WHO (CIDI-DSM-IV) scale for psychological outcomes at age 21	None	Yes	48%	Yes	Yes	Yes	No	Yes
**Mills et al. (2014)**	(Waves 1–4 = 168 months)	None	1. Smoking status; 2. Alcohol use	Yes	28%	Yes	No	Yes	No	Yes
**Mills et al. (2019)**	(Waves 1–5 = 252 months)	Peabody Picture Vocabulary Test (PPVT)	1. Failure to complete high school; 2. Failure to be employed or education at 21 years	No	48%	Yes	No	No	No	No
**Mills et al. (2017)**	(Waves 1–5 = 252 months)	1. WHO (CIDI-DSM-IV) scale for Cannabis use/ dependence; 2. Achenbach Child Behaviour Checklist (CBCL); 3. Delusions–Symptoms–States Inventory (DSSI)	Self-report	Yes	48%	Yes	No	No	No	No
**Author (Year)**	**Timeframe between reported waves (months)**	**Outcome Measures**		**Missing data (Yes/ No)**	**Attrition rate**	**Described attrition (Yes/No)**	**Corrected attrition (Yes/No)**	**Attrition analysis (Yes/No)**	**Selection bias (Yes/ No)**	**Sensitivity analysis Yes/No)**
		**Standardized**	**Non-standardized**							
**Parrish et al. (2011)**	(Waves 1–2 = 48 months)	None	Protective service report	No	22%	No	No	No	No	No
**Raghavan et al. (2012)**	Wave 1- Wave 4 = 48 months	Internalizing or externalizing scales of the CBCL	1. Non-zero Medicaid expenditures in a calendar year; 2. Mean total annual Medicaid expenditure per child	No	NR	No	No	No	No	Yes

Notes

**CIDI** Composite International Diagnostic Interview

**CPC** Child Protection Contact

**CPS** Child Protective Services

**CSA** Child Sexual Abuse

**DSM-IV** Diagnostic and Statistical Manual of Mental Disorders, 4^th^ edition

**DVSA** Domestic violence and sexual assault;

**IPV** Intimate Partner Violence

**LTFC** Long Term Foster Care

**N/A** Not Applicable

**NR** Not Reported

**OHC** Out-of-home care

**SDQ** Strength and Difficulties Questionnaire

**WHO** World Health Organisation

**YASR** Young Adult Self Report

## References

[1] Chikwava F, Cordier R, Ferrante A, O’Donnell M, Speyer R, Parsons L (2021) Research using population-based administration data integrated with longitudinal data in child protection settings: A systematic review. PLoS ONE 16(3): e0249088. 10.1371/journal.pone.0249088 33760881 PMC7990188

