# Supplementary material for: Research using population-based administration data integrated with longitudinal data in child protection settings: A systematic review
Source: PLoS One. 2021 Mar 24;16(3):e0249088. doi: 10.1371/journal.pone.0249088 (PMC7990188; doi:10.1371/journal.pone.0249088)
Supplement: S2 Table — (DOCX) [file pone.0249088.s002.docx]

**S2 Table. Search Strategy from all Databases**

| **Database and search terms** | **Number of records** |
| --- | --- |
| **CINAHL:**  ("data link*" or "record link*" or "linked data" or "linked record*" or "medical data" or "medical record*" or "electronic health data" or "electronic health record*" or "subject link*" or "subject-link*" or "population-based data" or "population based data" or "population-based record*" or "population based record*" or "population data" or "population record*" or "hospital data" or "hospital record*" or "personal data" or "personal record*" or "administrative data" or "administrative record*" or "cross-sectoral-link*" or "cross-sectoral link*" or "cross sectoral link*" or "cross-jurisdictional-link*" or "cross-jurisdictional link*" or "cross jurisdictional link*" or "cross-agency-link*" or "cross-agency link*" or "cross agency link*" or "census*" or "entity resolution*" or "deterministic link*" or "probabilistic link*" or "register*" or "registry" or "registries" or "personal data" or "personal record*") AND ("cohort stud*" or "longitudinal stud*" or "panel stud*" or "repeated measure*" or "prospective stud*" or "follow up stud*" or "follow-up stud*") AND ("out-of-home care" or "out of home care" or "out-of-home placement*" or "out of home placement*" or "foster care" or " foster home*" or "foster youth" or "child*-in care" or "child* in care" or "young people in care" or "adolescents in care" or "children’s home*" or "kinship" or "group home*" or "adopted child*" or "relative care" or "home-based care" or "home based care" or "orphan*" or "state care" or "public care" or "looked after child*" or "looked-after child*" or "substitute care" or "childcare system" or "child protection" or “child welfare" or “care leav*" or "care-leav*" or "leaving care" or "leaving-care" or "left care" or "transition* care" or "transition* from care") | 243 |
| **Embase: As per CINAHL.** | 415 |
| **Eric: As per CINAHL.** | 12 |
| **Medline: As per CINAHL.** | 311 |
| **PsycINFO: As per CINAHL.** | 142 |
