# Supplementary material for: Research using population-based administration data integrated with longitudinal data in child protection settings: A systematic review
Source: PLoS One. 2021 Mar 24;16(3):e0249088. doi: 10.1371/journal.pone.0249088 (PMC7990188; doi:10.1371/journal.pone.0249088)
Supplement: S3 Table — (DOCX) [file pone.0249088.s003.docx]

**S3 Table. Data Preparation Methods**

| **Statistical Analysis Domain** | **Statistical Analysis Procedure** | **Statistical parameters** | **N** | **%** |
| --- | --- | --- | --- | --- |
| Descriptive Analysis | | Frequencies, %, mean (sd), incidence rate, population attributable risk (PAR)% | 9 | 30% |
| Non-parametric test | Chi-squared test | Frequencies, percentages, Chi-squared statistic, chi-squared p-value | 16 | 53% |
| Parametric test | Two-sample t-test | Frequencies (95% CI), Percentages, F-test, t-statistics, significance level, Mean scores, standard errors, P-value, | 4 | 13% |
| Bivariate Analysis | Correlation Analysis | Pearson's coefficient | 2 | 7% |
|  | Logistic Regression | Frequencies, percentages, odds ratios (95% CI) | 1 | 3% |
|  | Concordance Analysis | Frequencies and percentages | 1 | 3% |
| Cumulative Risk Factor Analysis | | Sensitivity (%), Specificity (%), Positive Predictive value (PPV) | 1 | 3% |
| Attrition Analysis | Propensity Analysis | Odds ratios (95% CI), p-values | 3 | 10% |
|  | Inverse Probability Weighting-using Logistic Regression | Prevalence, Odds ratios (95% CI), p-values | 7 | 23% |
| Multiple imputation Methods | Markov chain iterative regression methods | NR | 1 | 3% |
|  | Chained equations analysis | % missing values, frequencies before/ after imputation) (95% CI) | 1 | 3% |
|  | Fully conditional specification method | NR | 1 | 3% |
| Sensitivity Analysis | Logistic Regression | Prevalence, Odds ratios (95% CI), p-values | 7 | 23% |
|  | Multiple Regression Analysis | Mean scores, regression coefficients, 95% CI | 1 | 3% |
