# Supplementary material for: Research using population-based administration data integrated with longitudinal data in child protection settings: A systematic review
Source: PLoS One. 2021 Mar 24;16(3):e0249088. doi: 10.1371/journal.pone.0249088 (PMC7990188; doi:10.1371/journal.pone.0249088)
Supplement: S1 File — (PDF) [file pone.0249088.s004.pdf]

A systematic review Protocol on research using population-based administration data linked to longitudinal surveys in out-of-home care or child protection settings

1. Introduction

Over the past decade, there has been a marked growth in the use of linked administrative data for child protection research to understand longitudinally, factors associated with outcomes in adulthood. Linked data allows researchers to analyse sensitive topics without directly asking study participants; however, administrative data is not collected for research purposes. Utilising administrative data linked to longitudinal surveys provides rich data allowing for more in-depth analysis and reduction in biases which may be present in both data sources. There is no systematic review that has been conducted to investigate the reporting of outcomes in children who have child protection involvement from studies where administrative dataset has been linked to longitudinal surveys.

2. Objectives

The primary objectives of the systematic review are:

1. To synthesise and describe the different research designs reported when integrating administrative data to longitudinal surveys in out-of-home care or child protection settings
2. To describe and assess the statistical methods used when retrospective administrative data is linked to prospective longitudinal surveys.

We will discuss the suitability of the methods identified in the included studies. The aim is not to document methods of the data linkage process, but rather provide evidence on the different ways in which linked data can be used to enhance survey data thereby minimising risk of bias and other limitations reported.

The findings from this review will enhance our understanding, applications and enhanced use of data from linking multiple administrative databases and self-ported survey measures to understand various measures and associations in multiple domains. This systematic review is an essential step towards informing policy, practice and future research directions in methodological aspects of using linked data and survey data.

Although research on linked administrative data and longitudinal surveys in child protection/ out-of-home care settings is abundant, to the best of our knowledge, no systematic reviews have documented integration of administrative data and prospective longitudinal surveys. In addition, no reviews have reported on the statistical methods used for integration and reporting on outcomes associated with combining the two data sources.

3. Methods

The methodology and reporting on this systematic review complies with the Preferred Reporting Items for Systematic Reviews and Meta-Analyses (PRISMA) statement and checklist. This is designed to guide researchers in evidence based and transparent reporting of systematic reviews.

3.1 Eligibility Criteria

To be included in this review, peer reviewed journal articles needed to have at least one administrative databases linked to longitudinal survey. The studies would be limited to those where contact with child protection services or out-of-home care settings is involved. They will also be limited to studies in the English-language only, and no restriction on the publication dates. In addition, since the review is focussed on methodology there will not be any reporting of specific interventions, comparison groups or outcomes. Articles involving systematic reviews will be excluded. In addition, anecdotes, reviews, book chapters, letters to the editor, editorials and conference abstracts will be excluded. Articles would have to meet all eligibility criteria to be included. See below table of inclusion and exclusion criteria:

Table 1: Inclusion and Exclusion Criteria

Inclusion Criteria	Exclusion Criteria
• Includes at least one administrative datasets<br>• links administrative data to longitudinal survey data<br>• Peer reviewed journal articles<br>• Human study population	• Papers ONLY utilising administrative data OR longitudinal data<br>• Non-peer reviewed articles: (Anecdotes, reviews, book chapters, letters to the editor, editorials and conference proceedings<br>• Unpublished, not available online<br>• Articles published in any language other than English<br>• Findings will not be included if only an abstract could be found.<br>• Reviews including systematic reviews are excluded.

3.2 Information sources

Research articles were searched from Medline (Ovid), Embase, ERIC, CINAHL and Pscyinfo databases. In addition, a search was conducted on some websites which provide a publication repository where data linkage units may list peer-reviewed publications, such as the Population Health Research Network (PHRN). The reference list of included studies was manually scrutinised to find any relevant studies. This provided an invaluable source of articles for inclusion. Searches were conducted using free-text in all databases.

3.3 Search Strategy

In line with the objectives of this review, three strings were used to search for articles in the relevant journals, and these include the following:

1. Data Source
2. Longitudinal Survey/ Study
3. “Out-of home care” or “child protection”

A full search strategy for all databases is attached.

3.4 Study Selection

Two review authors independently conducted screening of titles and abstracts of the retrieved studies to identify the candidate studies for the full text review. The second reviewer reviewed a randomly selected 40% of the abstracts to ensure accuracy in study selection for the review. The reviewers graded each article as eligible/ not eligible/ might be eligible (using the inclusion and exclusion criteria defined above). There were no disagreements on the eligibility for the inclusion of particular studies. Both reviewers were involved independently in full text review, data retrieving and quality assessment of the included studies.

3.5 Data collection process

Using a standardised form, the two reviewers will extract the data independently. A third reviewer will independently check the data for consistency and clarity.

3.6 Data Items

Data extracted will include the following broad summary measures: study/ population characteristics, population/ sample size, measurement scales, data collection tools, validation methods and analysis method. Specific variables that will be extracted include the following:

Study/ Population characteristics: Citation, year of publication, aims/ objectives, research area, study location, out-of-home care or child protection setting
Administrative Data: Source & name of administrative data, Sample/ population size of data, number of administrative datasets, linkage type and linkage quality
Longitudinal Survey data: Name of study, study duration, sampling method, study population (Age at baseline, gender, cohort size at baseline. Number of waves reported and sample sizes for each, attrition rate, standardised and non-standardised outcome measures, timeframe between waves, biases reported, and sensitivity analysis
Statistical Methods: Domain of statistical analysis, analysis procedure, statistical parameters of reporting, tests for assumptions, confounding variables, mediating/ moderating variables, outcome variables, independent variables.

3.7 Methodological Quality and risk of bias

Since there is no standard criteria for assessing the quality of this unique integration of population-based administrative data and longitudinal studies, we used a combination of methods for assessing the quality of studies. A combination of the terms from the RECORD checklist, the Guild Guidance and the Kmet checklist were used. Kmet checklist has 14 items which use a 3-point ordinal scale of (0 = no, 1 = partial, 2 = yes), giving a structure and quantifiable means for assessing the quality of studies of a variety of research designs. The checklist items assess the sampling strategy, participant characteristics described, sample size calculations, sample size collection, description and justification of analytic methods, result reporting, controls for confounding variables, and whether conclusions drawn reflect results reported. Interrater reliability between the two independent assessors will be established for both the abstract selection and Kmet ratings of each included study. The following convention will be used for the classification of methodological quality: a score of >80% is considered strong quality, a score of 70±79% is good quality, 50 ±69% is considered fair quality and <50% is considered to have poor methodological quality. The Guild and Record items will assess method of data linkage, if linkage quality and accuracy is reported and representativeness of linked data.

3.8 Data Synthesis

It is anticipated that the included studies will be heterogeneous in terms of study designs and quality of the studies. Therefore, narrative synthesis of the findings of the included studies will be an appropriate strategy. The synthesis will be structured to report the findings of each study on the data abstraction items defined above. If there are a number of studies that are homogeneous in terms of study design, quality, and outcome measures, and the number is substantial enough to conduct meta-analysis, the team will pool the results using the random-effects meta-analysis, with standardised mean differences for continuous outcomes and risk ratios for binary outcomes, and calculate 95% confidence intervals and two sided P values for each outcome.

References

1. Benchimol EI, Smeeth L, Guttmann A, Harron K, Moher D, Petersen I, et al. (2015) The REporting of studies Conducted using Observational Routinely-collected health Data (RECORD) Statement. PLoS Med 12(10): e1001885. doi:10.1371/journal.pmed.1001885
2. Gilbert, R., et al., GUILD: GUidance for Information about Linking Data sets. Journal of public health (Oxford, England), 2018. 40(1): p. 191-198.
3. Holman, C.D., et al., A decade of data linkage in Western Australia: strategic design, applications and benefits of the WA data linkage system. Australian Health Review, 2008. 32(4): p. 766-77.
4. Calderwood, L. and C. Lessof, Enhancing Longitudinal Surveys by Linking to Administrative Data. 2009. p. 55-72.
5. Young, A. and F. Flack, Recent trends in the use of linked data in Australia. Australian health review : a publication of the Australian Hospital Association, 2018. 42(5): p. 584-590.
6. Brownell, M.D. and D.P. Jutte, Administrative data linkage as a tool for child maltreatment research. Child Abuse & Neglect, 2013. 37(2): p. 120-124.
7. Tew, M., et al., Growth of linked hospital data use in Australia: a systematic review. Australian health review : a publication of the Australian Hospital Association, 2017. 41(4): p. 394-400.
8. Maclean, M.J., C.L. Taylor, and M. O’Donnell, Relationship between out-of-home care placement history characteristics and educational achievement: A population level linked data study. Child Abuse & Neglect, 2017. 70: p. 146.
9. Maclean, M.J., C.L. Taylor, and M. O'Donnell, Out-of-Home Care and the Educational Achievement, Attendance, and Suspensions of Maltreated Children: A Propensity-Matched Study. The Journal of pediatrics, 2018. 198: p. 287-293.e2.
10. Randall, S., et al., Sociodemographic differences in linkage error: an examination of four large-scale datasets. BMC health services research, 2018. 18(1): p. 678.
11. Ferrante, A., The Use of Data-Linkage Methods in Criminal Justice Research: A Commentary on Progress, Problems and Future Possibilities. Current Issues in Criminal Justice, 2009. 20: p. 378.
12. Hilder, L., et al., Preparing linked population data for research: cohort study of prisoner perinatal health outcomes. BMC Medical Research Methodology, 2016. 16.
13. Harron, K., et al., Challenges in administrative data linkage for research. Big Data & Society, 2017. 4(2): p. 2053951717745678.
14. Findlay, L., et al., Longitudinal child data: What can be gained by linking administrative data and cohort data? Vol. 3. 2018.
15. Carr, V.J., et al., New South Wales Child Development Study (NSW-CDS): an Australian multiagency, multigenerational, longitudinal record linkage study. BMJ Open, 2016. 6(2): p. e009023.
16. Simon, A., Using administrative data for constructing sampling frames and replacing data collected through surveys. International Journal of Social Research Methodology, 2014. 17(3): p. 185-196.
17. Cohen, S., et al., Administrative health databases for addressing emerging issues in adults with CHD: a systematic review. Cardiology in the Young, 2018. 28(6): p. 844-853.
18. Doiron, D.M.P.P., P.P. Raina, and I.P. Fortier, Linking Canadian Population Health Data: Maximizing the Potential of Cohort and Administrative Data. Canadian Journal of Public Health, 2013. 104(3): p. e258-61.
19. Mills, R., et al., Child Maltreatment and Adolescent Mental Health Problems in a Large Birth Cohort. Child Abuse & Neglect: The International Journal, 2013. 37(5): p. 292-302.
20. Meer, J. and N. Mittag, Using Linked Survey and Administrative Data to Better Measure Income: Implications for Poverty, Program Effectiveness and Holes in the Safety Net. 2015, W E Upjohn Institute for Employment Research.
21. Bell, M.F., et al., Using linked data to investigate developmental vulnerabilities in children of convicted parents. Developmental Psychology, 2018. 54(7): p. 1219-1231.
22. Eyawo, O., et al., Cohort profile: the Comparative Outcomes And Service Utilization Trends (COAST) Study among people living with and without HIV in British Columbia, Canada. BMJ Open, 2018. 8(1).

23. Parrish, J.W., et al., Quantifying sources of bias in longitudinal data linkage studies of child abuse and neglect: measuring impact of outcome specification, linkage error, and partial cohort follow-up. Injury Epidemiology, 2017. 4(1): p. 1-13.
24. Wagner, M., et al., The Special Education Elementary Longitudinal Study and the National Longitudinal Transition Study: Study Designs and Implications for Children and Youth With Emotional Disturbance. Journal of Emotional and Behavioral Disorders, 2005. 13(1): p. 25-41.
25. Connelly, L.M., Cross-Sectional Survey Research. Medsurg Nursing, 2016. 25(5): p. 369-370.
26. Knies, G., J. Burton, and E. Sala, Consenting to health record linkage: evidence from a multi-purpose longitudinal survey of a general population. BMC Health Services Research, 2012. 12: p. 52.
27. Mills, R., et al., Self-reported and agency-notified child sexual abuse in a population-based birth cohort. Journal of psychiatric research, 2016. 74: p. 87-93.
28. Jutte, D.P., L.L. Roos, and M.D. Brownell, Administrative record linkage as a tool for public health research. Annual review of public health, 2011. 32: p. 91-108.
29. Macdonald, K.I., S.J. Kilty, and C. van Walraven, Chronic rhinosinusitis identification in administrative databases and health surveys: A systematic review. The Laryngoscope, 2016. 126(6): p. 1303-1310.
30. Leong, A., et al., Systematic Review and Meta-Analysis of Validation Studies on a Diabetes Case Definition from Health Administrative Records: e75256. PLoS ONE, 2013. 8(10).
31. van Mourik, M.S.M., et al., Accuracy of administrative data for surveillance of healthcare-associated infections: a systematic review. BMJ open, 2015. 5(8): p. 1.
32. Farzanfar, D., et al., Longitudinal studies that use data collected as part of usual care risk reporting biased results: a systematic review. BMC Medical Research Methodology, 2017. 17(1): p. 133.
33. Karahalios, A., et al., A review of the reporting and handling of missing data in cohort studies with repeated assessment of exposure measures. BMC medical research methodology, 2012. 12: p. 96.
34. Andrade, D.d., et al., The effectiveness of residential treatment services for individuals with substance use disorders: A systematic review. Drug and Alcohol Dependence, 2019. 201: p. 227.
35. da Silva, M.E., et al., Informed consent for record linkage: a systematic review. Journal of Medical Ethics, 2012. 38(10): p. 639-42.
36. Moher, D., et al., Preferred reporting items for systematic review and meta-analysis protocols (PRISMA-P) 2015 statement. Systematic Reviews, 2015. 4.
37. Kmet, L.M., L.S. Cook, and R.C. Lee, Standard Quality Assessment Criteria for Evaluating Primary Research Papers from a Variety of Fields. Edmonton: Alberta Heritage Foundation for Medical Research (AHFMR), 2004. AHFMR - HTA Initiative #13.
