# Supplementary material for: Research using population-based administration data integrated with longitudinal data in child protection settings: A systematic review
Source: PLoS One. 2021 Mar 24;16(3):e0249088. doi: 10.1371/journal.pone.0249088 (PMC7990188; doi:10.1371/journal.pone.0249088)
Supplement: Attachment — Submitted filename: Response to Reviewers.docx [file pone.0249088.s005.docx]

To PLOS One

Reviewers and Academic Editors

Dear Sir/ Madam

**Re: Response to reviewers**

We would like to thank the Reviewers and Editors for the generous comments on the manuscript and have edited the manuscript to address their concerns. The responses are as follows:

**Comments from Reviewer #1:** *“I found the review inspiring and enjoyed reading it.
One of the main results (lack of information on data linkage process and quality) clearly is important for future studies. What seems to be missing is an analysis and discussion on quality criteria for administrative data sets as we and others have shown that there are huge differences (e.g. reliability of substantiation process for child maltreatment notifications)”*

**Response:** This is an important point raised by the reviewer and we have added in an additional recommendation in the manuscript to address this issue with four additional references:

**Additional text in manuscript: (Lines 797-816)**

“In addition, our review has also shown that there was lack of reporting or referencing of validated data quality assessments conducted for administrative data. In the context of transparency, accuracy, and reliability of measurement from administrative data sources, it is important to reference validated appraisal tools. Additionally, due to variability in quality criteria for child protection administrative data sets, we recommend that future researchers implement a data quality framework [130, 131]. With the growing use of administrative data it is necessary that data quality indicators are operationalised and reported in studies. For example, leaders in the use of linked administrative data at the Manitoba Centre of Health Policy have identified 5 dimensions of data quality: accuracy, internal validity, external validity, timeliness, and interoperability.

These dimensions of data quality can serve as an important starting point for future reporting of administrative data. However, determining if these dimensions are comprehensive, what exact criteria should be used for each dimension, and the operationalisation of those dimensions into measurable data quality criteria remains elusive. As such, there is need to conduct a Delphi Study [132, 133] among leading experts in the field of administrative data, to establish consensus on the use of these data quality indicators to either be integrated into tools such as the GUILD [7] and RECORD [79] guidelines, or to develop a new comprehensive data quality appraisal tool.”

**Additional References:**

1.	Laitila T, Wallgren A, Britt, Wallgren B. Quality Assessment of Administrative Data. 2011.

2.	Smith M, Lix LM, Azimaee M, Enns JE, Orr J, Hong S, et al. Assessing the quality of administrative data for research: a framework from the Manitoba Centre for Health Policy. Journal of the American Medical Informatics Association : JAMIA. 2018;25(3):224-9. doi: http://dx.doi.org/10.1093/jamia/ocx078. PubMed PMID: 1988263660; 29025002.

3.	Chia-Chien H, Sandford BA. The Delphi Technique: Making Sense of Consensus. Practical Assessment, Research & Evaluation. 2007;12:10. PubMed PMID: 2366822829.

4.	Grisham T. The Delphi technique: a method for testing complex and multifaceted topics. International Journal of Managing Projects in Business. 2009;2(1):112-30. doi: http://dx.doi.org/10.1108/17538370910930545. PubMed PMID: 232630764.

**Comments from Reviewer #2: *“****For future studies, the researcher can expand his/her inclusion criteria and geographic scope, assessing peer reviewed and non-peer reviewed publications, published in languages other than English and regions including Africa and Asia.”*

**Response:** We have added an additional point in the limitation section:

**Additional text in manuscript: (Lines 770 - 773):**

“Future reviews should consider targeted searches that may uncover literature from other geographic regions such as Asia, Africa and South and Central America.”

The authors hope that the Reviewers and Editors will be satisfied with the further amendments which we have made to the manuscript which we feel has strengthened the paper.
